# Immediate effect of inspiratory exercise with exerciser and respiratory encourager in women without vocal complaints

**DOI:** 10.1590/2317-1782/20242023148en

**Published:** 2024-05-20

**Authors:** Alessandra Thais Beraldo, Julia Batistella, Perla do Nascimento Martins, Ana Paula Dassie-Leite, Eliane Cristina Pereira

**Affiliations:** 1 Departamento de Fonoaudiologia, Universidade Estadual do Centro-Oeste – UNICENTRO - Irati (PR), Brasil.

**Keywords:** Voice, Respiration, Breathing Exercises, Voice Quality, Voice Training

## Abstract

**Purpose:**

To evaluate the immediate effect of the inspiratory exercise with a booster and a respiratory exerciser on the voice of women without vocal complaints.

**Methods:**

25 women with no vocal complaints, between 18 and 34 years old, with a score of 1 on the Vocal Disorder Screening Index (ITDV) participated. Data collection was performed before and after performing the inspiratory exercise and consisted of recording the sustained vowel /a/, connected speech and maximum phonatory times (MPT) of vowels, fricative phonemes and counting numbers. In the auditory-perceptual judgment, the Vocal Deviation Scale (VSD) was used to verify the general degree of vocal deviation. Acoustic evaluation was performed using the PRAAT software and the parameters fundamental frequency (f0), jitter, shimmer, harmonium-to-noise ratio (HNR), Cepstral Peak Prominence Smoothed (CPPS), Acoustic Voice Quality Index (AVQI) and Acoustic Breathiness Index (ABI). To measure the aerodynamic measurements, the time of each emission was extracted in the Audacity program. Data were statistically analyzed using the Statistica for Windows software and normality was tested using the Shapiro-Wilk test. To compare the results, Student's and Wilcoxon's t tests were applied, adopting a significance level of 5%.

**Results:**

There were no significant differences between the results of the JPA and the acoustic measures, in the pre and post inspiratory exercise moments. As for the aerodynamic measures, it was possible to observe a significant increase in the value of the TMF /s/ (p=0.008).

**Conclusion:**

There was no change in vocal quality after the inspiratory exercise with stimulator and respiratory exerciser, but an increase in the MPT of the phoneme /s/ was observed after the exercise.

## INTRODUCTION

The respiratory system plays a vital role in the exchange of gases between the body and the environment. However, it is also essential for voice production. This is because this system works like a pump, in which the airflow and air pressure promote the activation of the vibratory structures of the vocal folds (VFs) and, consequently, the emission of speech sounds^(1)^.

According to the myoelastic-aerodynamic theory, during phonation, when expiratory airflow begins, glottal closure causes the subglottic air pressure to exceed the supraglottic pressure. Consequently, the subglottic pressure pushes the lower edges of the vocal folds apart, initiating glottal opening. After opening, the elasticity of the vocal fold tissues will force the vocal folds to reverse the movement from opening to closing^(2)^.

In this way, training and proper use of the respiratory muscles influence efficient vocal production, as it provides the necessary airflow for adequate subglottic pressure, essential for vocal fold vibration. There are a variety of treatment approaches for training the respiratory muscles in the vocal clinic, but their effects on aerodynamic measurements are still scarce^(3)^.

Seeking to improve phonatory efficiency, speech-language pathologists have been utilizing respiratory trainers and stimulators to work on the inspiratory and expiratory muscles, in addition to specific work on the laryngeal muscles^(4-9)^. Such resources provide increased lung capacity, generating greater activation of the diaphragm and intercostal muscles^(10)^.

Respiratory trainers and stimulators are devices that stimulate breathing based on muscle effort. In the case of inspiratory exercises with breathing trainers and stimulators, they encourage deep inspiration, with maximal lung expansion and, thus, assist in muscular activity, mainly in the diaphragm and other intercostal muscles responsible for respiratory movement. Since these are muscles, they can be trained to improve strength and resistance to fatigue^(11)^.

The literature indicates that inspiratory training with respiratory trainers and stimulators is capable of increasing maximum phonation times and air storage capacity^(4)^. The increase in respiratory muscle strength from training with stimulators has also been described; However, its effects on aerodynamic measurements and vocal characteristics of the subjects are still unclear^(5)^. A recent systematic review study concluded that there is still a lack of evidence regarding the effectiveness of respiratory interventions on vocal results, emphasizing the importance of understanding the mechanism of action of exercises and their potential impacts on voice production^(10)^.

During vocal production, the muscles involved in breathing work together to provide the airflow necessary to produce sound. It is believed that studies on respiratory training, as well as its effects on the voice, are promising for the Speech Therapy Clinic. This research aims to clarify the effects of inspiratory muscle training with a respiratory trainer and stimulator on the voice in order to evaluate the relevance or feasibility of its introduction into the rehabilitation process of vocal disorders or improvement of professional voices.

Starting from the hypothesis that inspiratory exercises with respiratory trainers and stimulators could positively impact the voice, the objective of this study was to evaluate the immediate effect of the inspiratory exercise with a respiratory trainer and stimulator on the perceptual-auditory, acoustic, and aerodynamic data of women without vocal complaints.

## METHOD

This is a pre-post intervention study.

The research was approved by the Ethics Committee on Human Research with protocol number 5,594,862.

### Casuistry

The sample was constituted by convenience, and the target population of the study was a group consisting of women without vocal complaints. The subjects were their own controls.

Data collection took place in the Voice Laboratory of the home institution, at times previously established with the women participating in the research.

#### Inclusion criteria

Women without vocal complaints were included, aged over 18 years, undergraduate students, who voluntarily agreed to participate in the research stages and signed the Informed Consent Form (ICF).

#### Exclusion criteria

Women with vocal complaints, who presented a score equal to or greater than 5 on the Voice Disorder Screening Index (VDSI)^(12)^, smokers, who undergo or have undergone vocal treatment or laryngeal surgery, who self-reported hearing loss, with previous hormonal, gastric, pulmonary, neurological, or psychiatric diseases, who presented signs and symptoms of airway changes on the day of research data collection, and those who did not take part in any stage of the the research.

### Sample calculation

The sample size calculation was carried out by testing hypotheses for the Two Proportions Test based on a similar study that analyzed the effect of the Shaker Plus respiratory stimulant before and after three minutes of exercise. The fixed parameters adopted for the test were α of 5%, β of 20%, and K of 80%.

α: Error α (Significance level) is the probability of being wrong in accepting the Alternative Hypothesis (H1) when using a hypothesis test in a data analysis.

β: Error β is the probability of being wrong in rejecting the Alternative Hypothesis (H1) when using a hypothesis test in data analysis.

K (n pre/n post): It is the desired sample size proportion between the pre and post groups.

The proportions of improvement and worsening of the overall degree of vocal quality between the moments before and after three minutes of intervention were compared for the counting sample to estimate the variability of the intervention. The proportion of worsening (p1=0.43) and proportion of improvement (p2=0.07) values were used. The calculated sample size was 20 participants in the pre and post groups.

### Sample

Data was collected from 28 women. Of these, 3 were excluded because they had a score equal to or greater than 5 on the Voice Disorder Screening Index (VDSI)^(12)^.

Therefore, 25 women participated in the study, aged between 18 and 34 years old, with an average of 21.04 years old. The VDSI of the women included in the study had an average score of 1 point, a minimum of 0 and a maximum of 3 points. Regarding vocal characterization, the participants had an overall average vocal deviation of 40 points in vowel emission and 32 points in number emission (VAS). They also had an average F_0_ of 214Hz and median jitter% values of 0.4, shimmer% 2.58, HRN 20.93, CPPS 14.81, AVQI 1,68, and ABI 2,92.

### Procedures performed

The following procedures were performed:

1. Signing of the Free and Informed Consent Form;
2. Filling out a questionnaire about identification data and vocal complaints;
3. Application of the Voice Disorder Screening Index (VDSI)^12^ protocol.
4. Recording of the following vocal samples^(1)^:
  a. Sustained vowel /a/;
  b. Counting numbers from 1 to 20;
  c. MPT: sustained emissions of /a/, /i/, /u/, /s/, /z/, and counting numbers in a single exhalation;

Vocal samples were collected in a voice laboratory using a unidirectional microphone from the brand *Shure* model SM58 with an M Audio Fast Track audio interface, positioned in front of the mouth at an approximate distance of five centimeters. All participants were instructed to remain seated, with their torso upright, their back resting on the chair, arms relaxed, hands resting on their legs, and feet flat on the floor. The recording was made using the software Audacity at a sampling rate of 44,100 Hz, mono channel at 16 Bits.

Through voice recording, auditory-perceptual judgment (APJ), vocal acoustic analysis, and aerodynamic measurements of the emission were carried out.

The participants were instructed to emit at their usual frequency and intensity. Although the sound pressure was not controlled by measuring in decibels (dB), the audio input window was monitored during the recording so that the signal filled the entire range between -0.5 and 0.5, without exceeding that range, avoiding saturation.

Respiratory training using an inspiratory stimulator was carried out as follows:

d. Perform a series of 30 repetitions while inhaling with the Respiron® Classic Breathing Trainer and Stimulator^(13)^, following the guidelines below:

i. Inhale through your mouth until you elevate the three spheres of the Respiron® Classic, keeping them elevated for as long as possible, taking care not to elevate the accessory muscles excessively.

e. The exercise was performed in the position corresponding to zero effort, which has a graduation level of zero, one, two, and three.

Immediately after performing the exercise with the respiratory trainer and stimulator, the voice recording was repeated according to step 4.

### Auditory-perceptual judgment

The auditory-perceptual judgment (APJ) analyzed the general degree of vocal deviation of the sustained vowel /a/ and the counting of numbers from 1 to 11 using the Vocal Deviation Scale^(14)^. A 100-point visual analogue scale (VAS) was used. The end on the left means no deviation, and the end on the right means maximum deviation^(14)^.

The judge was a speech therapist specialized in voice, with 20 years of clinical experience. They received a folder with the voices in pairs, without knowing which was the pre moment and which was the post moment. The judge’s internal agreement was analyzed using the Intraclass Correlation Coefficient (ICC) statistical test, repeating 20% of the sample, randomly. After analysis by the ICC, it was found that the pairs were in agreement with each other, all answers above 0.9, which determines excellent agreement.

### Acoustic voice analysis

The software PRAAT was used to carry out the vocal acoustic analysis, and the following measurements were extracted by the emission of the sustained vowel /a/:

- Average fundamental frequency (F_0_): corresponds to the number of glottal cycles produced by the vocal folds in one second^(1)^;
- *Jitter*: corresponds to the variability of F_0_ in the short term, considering the glottal cycle, which are in sequence with the other. It measures how much a cycle differs from its predecessor according to the frequency disturbance^(1)^;
- *Shimmer*: corresponds to the short-term amplitude disturbance, which indicates small variations in the control of the airflow by VFs and the intensity of the emission^(1)^;
- *Harmonic-to-Noise Ratio* (HNR): it provides complementary information between the relationship of the harmonic component and the amount of sound noises in the vocal sample^(1)^;
- *CPPS*: it measures the degree of periodicity of the vocal signal above the noises present in sustained vowel and connected speech emissions, providing results in decibels. The measure produces an eminent improvement in the accuracy of the analysis of deviated voices^(15)^;

The following measurements were also extracted through emission of the vowel /a/ associated with counting the numbers from 1 to 11:

- Acoustic Voice Quality Index (AVQI): it quantifies the intensity of deviation in vowel quality^(16,17)^;
- *Acoustic Breathiness Index* (ABI): it provides information for screening and monitoring the patient’s breathy vocal quality^(18)^.

### Aerodynamic emission measurements

The Audacity software was used to take the aerodynamic measurements. The measurements were analyzed by a researcher who did not take part in any of the previous stages of the study. The Maximum Phonation Time (MPT) was collected, a parameter with which respiratory measurements are obtained and allows a quantitative and qualitative investigation of phonation^(1,19)^. The following measurements were used:

- /a/, /i/, /u/, /s/, /z/, and counting numbers in a single exhalation^(1,19)^;
- s/z relationship – extracted from the sustained emission of the voiceless and voiced medial fricatives /s/ and /z/. From this, the proportion between them was made, dividing the time of /s/ by that of /z/. With this measurement, the relationship between pulmonary aerodynamic forces and laryngeal myoelastic forces^(1,19)^ was obtained.

The results were compared by analyzing samples from the records of the Study Group before performing the inspiratory exercise (SG1) and the Study Group after performing the exercise (SG2). These were arranged and used for statistical analysis in the search for answers to the following hypotheses:

H0: there are no differences between auditory-perceptual evaluation, acoustic vocal analysis, and aerodynamic measures of emission before and after immediate exercise with an inspiratory stimulator.

H1: there is no equality between the auditory-perceptual judgments acoustic vocal analysis and the aerodynamic measurements of emission before and after the immediate performance of the exercise with an inspiratory stimulator.

It is hypothesized that working with the respiratory muscles can improve aspects of the mucosal wave movement of the vocal folds by modifying the transglottic flow and subglottic pressure after inspiratory training. Therefore, the general degree of vocal deviation, acoustic, and aerodynamic measurements were selected for the multidimensional assessment of the voice after performing inspiratory exercise with a respiratory trainer and stimulator.

### Study variables

The dependent variables (endpoints) of the study are the auditory-perceptual evaluation, the acoustic, and the aerodynamic measurements of the emission. The independent variable was exercise with a respiratory trainer and stimulator.

### Data analysis

The results were arranged in a Microsoft Excel spreadsheet and subjected to statistical analysis.

Measures of central tendency and dispersion for continuous variables with symmetric distribution were expressed as means and standard deviation (mean + SD), and for those with asymmetric distribution in medians, minimum, and maximum values.

The data was analyzed using the *software Statistica for Windows*. The normality of the variables was tested using the Shapiro Wilk test (p<0.05), all of which had a normal distribution, except for the TMF /a/ measure, which had a non-normal distribution. To compare the results, the *Student* parametric t-test was used for dependent variables in measures with a normal distribution, and the Wilcoxon test for the only non-normal measure. For all tests, a minimum significance level of 5% (p > 0.05) was considered.

## RESULTS

Twenty five women participated in the study with an average age of 21.04 years, the minimum of 18 years, and the maximum of 34 years. All were university students.

Table 1 shows the results for the distribution of the auditory-perceptual judgment values for the general degree of vocal deviation before and after inspiratory exercise with a respiratory trainer and respiratory stimulator for the sustained vowel /a/ and the counting of the numbers. There were no differences between emissions before and after the exercise. It is important to mention that the mean values presented by the participants, both pre and post, were within normal variability for the number sample and as discrete deviations for the vowel sample^(14)^.

**Table 1:** Distribution of auditory-perceptual judgment values of the general degree of vocal deviation of the vowel /a/ and number counting before and after exercise with inspiratory stimulator (N=25)

		Average	Median	Min.	Max.	Standard deviation	P
**Vowel /a/**	Pre	40.00	39	23	53	9.34	0.654
	Post	40.76	40	25	56	9.43	
**Counting of Numbers**	Pre	32.00	32	10	54	12.32	0.221
	Post	29.28	29	13	51	10.73	

Student's t test for dependent variables

Table 2 shows the results for the distribution of acoustic values before and after inspiratory exercise with a respiratory trainer and respiratory stimulator. There were no differences between the analyses before and after performing the exercise.

**Table 2:** Distribution of acoustic values before and after performing the exercise with inspiratory stimulator (n=25)

		Average	Median	Min.	Max.	Standard deviation	P
**F0 (%)**	Pre	214.56	225.99	111.95	250.17	30.72	0.513
	Post	219.26	217.21	157.16	276.25	24.91	
**Jitter (%)**	Pre	0.40	0.40	0.18	0.60	0.12	0.890
	Post	0.44	0.34	0.17	0.99	0.21	
**Shimmer (%)**	Pre	2.58	2.44	1.12	5.72	0.92	0.566
	Post	2.46	2.13	1.04	5.25	1.09	
**Shimmer (dB)**	Pre	0.22	0.21	0.09	0.49	0.08	0.616
	Post	0.21	0.19	0.09	0.46	0.09	
**HNR (%)**	Pre	20.93	21.22	16.06	28.56	2.79	0.925
	Post	20.98	21.43	15.80	26.16	2.77	
**CPPS (%)**	Pre	14.81	14.58	12.29	17.87	1.49	0.322
	Post	15.09	15.31	12.15	17.65	1.59	
**AVQI (%)**	Pre	1.68	1.57	0.14	3.03	0.78	0.532
	Post	1.75	2.05	0.05	3.66	0.87	
**ABI (%)**	Pre	2.92	2.83	1.7	5.08	0.8	0.889
	Post	2.94	2.91	1.8	5.25	0.81	

Student's t test for dependent variables

Table 3 shows the results for the distribution of aerodynamic measurements before and after inspiratory exercise with a respiratory trainer and stimulator. There was a difference between the values of MPT /s/ before and after the inspiratory exercise.

**Table 3:** Distribution of values of maximum phonation times before and after exercise with inspiratory stimulator (n=25)

		Average	Median	Min.	Max.	Standard deviation	p
**MPT /a/**	Pre	14.12	13	10	28	4.35	0.930^1^
	Post	13.8	13	10	21	2.95	
**MPT /i/**	Pre	13.72	14	8	23	3.27	0.262
	Post	14.16	13	10	24	3.36	
**MPT /u/**	Pre	13.68	14	7	22	3.27	0.320
	Post	14.28	14	9	23	3.27	
**MPT /s/**	Pre	12.84	12	5	24	4.37	0.008^*^
	Post	14.56	15	6	22	4.26	
**MPT /z/**	Pre	13.72	13	6	28	4.70	0.348
	Post	14.40	13	8	25	4.67	
**MPT counting numbers**	Pre	17.04	16	10	29	4.72	0.232
	Post	17.76	17	11	27	4.24	
**S/z ratio**	Pre	0.971	0.93	0.52	1.72	0.307	0.183
	Post	1.037	1.0	0.64	1.70	0.266	

1 Student's t test for dependent variables;

Wilcoxon test

* p<0.05

## DISCUSSION

The Evaluation of the immediate effect of a vocal technique seeks to understand the physiological changes in the phonatory mechanism^(20)^. This study sought to investigate the immediate effects of inspiratory exercise with a respiratory trainer and respiratory stimulator in women with no vocal complaints. The choice of women without vocal complaints sought to understand the effect of the exercise primarily on vocally healthy women or those with mild vocal deviation in order to subsidize future studies on dysphonic subjects. This study also seeks evidence that can help the clinician select the most efficient techniques for immediate results. It is understood that the muscles involved in breathing work together to provide the airflow needed to produce the voice and that training these muscles can modify transglottic airflow and air pressure, generating vocal impact.

The sample selected for the study was made up of women with no vocal complaints who underwent voice screening by the SIVD^(12)^ and had their voice classified by auditory-perceptual judgment of the general degree of vocal deviation using the Vocal Deviation Scale (VDS)^(14)^. The classification of vocal deviation was compatible with that of healthy to slightly altered voices for counting numbers (between 0 and 35.5 mm)^(14)^ and mild to moderate for sustained vowel emission (between 35.5 mm and 50.5 mm)^(14)^, with no differences before and after the exercise with the inspiratory stimulator. The different classifications between the vocal samples collected, sustained vowel, and number counting, may have occurred due to the muscular adjustments required between source and filter in the different emissions. It is believed that the slight deviations in the emission of vowels do not compromise the results of the study since the average values were very close to the limit of normal variability. Furthermore, slight deviations are not uncommon in people without vocal complaints.

It was observed that performing the inspiratory exercise with a respiratory trainer and stimulator was not able to generate changes in vocal quality immediately afterward. In fact, this exercise has no direct laryngeal action. Therefore, inspiratory exercises with a respiratory trainer and stimulator should not be performed with the aim of achieving immediate changes in vocal quality, such as in vocal warm-up programs. Vocal techniques that have obtained positive responses in vocal quality in studies of immediate effects are more suitable for this purpose. However, it is believed that auditory-perceptual data should be further explored, especially in studies with longer respiratory training (weeks, months), based on the hypothesis that increases in subglottic pressure could have an effect on the coaptation and vibration of the vocal folds and, consequently, on voice stability.

As part of the multidimensional assessment of the voice, an acoustic analysis was carried out, which allows the properties of the vocal signal to be measured quantitatively, enabling an objective analysis of the voice^(21)^. Acoustic analysis is widely used in speech therapy vocal clinics through measurements of F0, *jitter, shimmer*, HNR *(Harmonic noise ratio*), CPPS, AVQI, among others^(16,17,22,23)^.

With regard to vocal acoustic analysis and auditory-perceptual evaluation, no differences were observed when comparing the moments before and after performing the inspiratory exercise with the respiratory trainer and stimulator. This exercise mainly works the respiratory muscles, such as the diaphragm and intercostal muscles.

There is the possibility that longer training will modify these parameters. The measure that varied the most was F_0_, with a decrease but not a significant one. This variation may be related to the lowering of the larynx that occurs during the exercise. More studies are needed to investigate whether this and other parameters can change when performing inspiratory exercises for longer periods. No studies were found that investigated acoustic measurements of voice related to exercises with an inspiratory stimulator.

Regarding the aerodynamic measurements evaluated through maximum phonation times, it was possible to observe significant changes in the MPT values of /s/ after performing the exercise with the inspiratory stimulator. The MPT is an acoustic measure capable of analyzing the pulmonary aerodynamic and myoelastic forces of the larynx^(1,24,25)^.

The phoneme /s/ is voiceless because it does not use the vibration of the vocal folds. With this measure, the aerodynamic control of the emission with the expiratory air exit can be demonstrated quantitatively, thus verifying the performance of the respiratory level in phonation^(1,19,24)^. This result shows that the practice of exercise with a respiratory stimulator can increase lung capacity.

The evidence of vocal treatment on maximum phonation time (MPT) was quantified using the statistical approach of a meta-analysis. Studies were considered that were reports of randomized controlled clinical trials (RCTs) evaluating the effectiveness of a specific speech therapy treatment using MPT as an outcome measurement in adult participants with voice disorders. The authors identified that the only effective intervention with a significant effect was vocal function exercise (VFE) and concluded that it effectively improved MPT from pre- to post-treatment compared to other comparative vocal interventions. The authors further suggest that more high-quality intervention studies with large sample sizes, multidimensional measurements, and homogeneous dysphonia groups are needed to support evidence-based practice in laryngology^(3)^.

The influence of body position and number of emissions on the results of maximum phonation times (MPT) of adults without voice complaints were analyzed in a previous study with sixty subjects, 30 men and 30 women without vocal complaints. The subjects were instructed to remain in an upright or sitting position, according to the order of collection selected. The first 30 subjects were evaluated in order number 1 (sitting and upright) and the other 30 subjects in order number 2 (upright and sitting), with a 5-minute interval between emissions in the two positions. The MPT of the vowels /a/, /i/, /u/, fricatives /s/ and /z/, and numbers were collected. Then, the subjects were instructed to perform the same emissions mentioned above but in another position (upright or sitting). There was no difference between the MPT obtained in the two positions. All males' MPTs were greater than those of females. Regarding the number of emissions, there were differences for both sexes in the MPT of the vowel /a/ and in the counting of numbers. The authors concluded that, in general, the body position does not influence the results of temporal voice measurements, but gender and the number of emissions influence the results of MPT^(19)^.

A study with five healthy adults identified that after training for four weeks with the Respiron® respiratory trainer, Classic Medium Level model, there was an improvement in the final MPT averages. The authors suggested that this happened because the use of respiratory trainers provides an increase in lung capacity. Therefore, the greater aerodynamic flow provided greater vocal control and emission capacity^(4)^.

Another study sought to determine and evaluate the effects of Diaphragmatic Breathing Exercise on respiratory function and vocal sustain among apparently healthy vocalists. The authors observed that the respiratory exercise had a direct effect on lung function and an indirect effect on the vocalists' maximum phonation time since there was a significant improvement in respiratory functions, and respiratory values significantly correlated with MPT values. They also found that changes in respiratory functions led to changes in maximum phonation time, as male singers in the research showed more changes in respiratory function than female singers, who showed better results in MPT^(25)^.

There are still few studies in the literature on the effects of inspiratory exercise with a respiratory trainer and stimulator on the voice. Other studies were found using the Respiron® Classic equipment, however, in an inverted way, to work the expiratory muscles. The respiratory trainer and stimulator “Respiron® Classic- NCS” was developed with the aim of strengthening the inspiratory muscles, with the diaphragm being the most important one. The instructions of the equipment do not recommend using the device inverted, and for this reason, such studies were not cited.

One limitation of this study was related to men's participation. As also evidenced in another study with breathing exercises, but with expiration, men's participation was difficult, which meant that the study was also carried out only with women^(8)^. Another limitation is the lack of self-assessment by the participants regarding the possible effects of the exercise on the voice. This procedure was not included because there are no self-assessment instruments available specifically for this purpose. In view of this, we understand that the application of existing self-assessment protocols, but with other purposes, would not provide relevant data for the present study. We highlight the importance of developing sensitive instruments to identify the subject/patient's vocal perception of the immediate effects of vocal techniques.

It is suggested that new studies with inspiratory exercises be carried out to test the immediate effects in different populations, such as men without vocal complaints, dysphonic women, and dysphonic men, as well as with other means of investigation, such as Spirometry for speech therapy purposes and with longer duration of use to check the effects of muscle training during consecutive weeks.

## CONCLUSION

The increase in the maximum phonation time of /s/ in women without vocal complaints demonstrates the immediate effectiveness of performing the inspiratory exercise with a breathing trainer and stimulator in lung capacity, demonstrating improvement in aerodynamic control with better respiratory performance during phonation.

In the other multidimensional vocal assessments carried out in this study, such as in the auditory-perceptual judgment and acoustic measures, the inspiratory exercise did not cause immediate changes.
