# Supplementary material for: Immediate effect of inspiratory exercise with exerciser and respiratory encourager in women without vocal complaints
Source: Codas. 2024 May 20;36(4):e20230148. doi: 10.1590/2317-1782/20242023148en (PMC11129852; doi:10.1590/2317-1782/20242023148en)
Supplement: Translation

# Translation: Efeito imediato de exercício inspiratório com exercitador e incentivador respiratório em mulheres sem queixas vocais

**DOI:** 10.1590/2317-1782/20242023148pt

**Authors:** Alessandra Thais Beraldo, Julia Batistella, Perla do Nascimento Martins, Ana Paula Dassie-Leite, Eliane Cristina Pereira

**Affiliations:** 1 Departamento de Fonoaudiologia, Universidade Estadual do Centro-Oeste – UNICENTRO - Irati (PR), Brasil.

**Keywords:** Voz, Respiração, Exercícios Respiratórios, Qualidade da Voz, Treinamento da Voz

## Abstract

**Objetivo:**

Avaliar o efeito imediato do exercício inspiratório com incentivador e exercitador respiratório na voz de mulheres sem queixas vocais.

**Método:**

Participaram 25 mulheres sem queixas vocais, entre 18 e 34 anos, com pontuação 1 no Índice de Triagem para Distúrbio Vocal (ITDV). A coleta de dados foi realizada nos momentos antes e após realização de exercício inspiratório e consistiu na gravação de vogal sustentada /a/, fala encadeada e tempos máximos fonatórios (TMF) de vogais, fonemas fricativos e contagem de números. No julgamento perceptivo-auditivo foi utilizada a Escala de Desvio Vocal (EDV) para verificar o grau geral do desvio vocal. Avaliação acústica foi feita no software PRAAT e foram extraídos os parâmetros frequência fundamental (f0), jitter, shimmer, proporção harmônico -ruído (HNR), Cepstral Peak Prominence Smoothed (CPPS), Acoustic Voice Quality Index (AVQI) e Acoustic Breathiness Index (ABI). Para mensuração das medidas aerodinâmicas, o tempo de emissão foi extraído no programa Audacity. Para comparar os resultados utilizou-se o teste paramétrico t de Student para amostras dependentes na análise das variáveis com distribuição normal e o teste de Wilcoxon para variáveis com distribuição não normal.

**Resultados:**

Não houve diferenças entre os resultados do JPA e das medidas acústicas, nos momentos pré e pós exercício inspiratório. Quanto às medidas aerodinâmicas foi possível observar aumento significativo no valor do TMF /s/ (p=0,008).

**Conclusão:**

Não houve modificação na qualidade vocal após o exercício inspiratório com incentivador e exercitador respiratório, porém foi observado aumento do TMF do fonema /s/ após a realização do exercício.

## INTRODUÇÃO

O sistema respiratório atua na função vital de trocas gasosas entre o organismo e meio ambiente, entretanto, também é essencial para a produção da voz. Isso porque esse sistema funciona como uma bomba, em que o fluxo e a pressão do ar promovem a ativação das estruturas vibratórias das pregas vocais e, consequentemente, a emissão dos sons da fala^(1)^.

De acordo com a teoria mioelástica-aerodinâmica, durante a fonação, quando o fluxo de ar expiratório se inicia, a coaptação glótica faz com que a pressão do ar subglótica seja maior do que a pressão supraglótica, assim sendo, a pressão subglótica afasta as margens inferiores das pregas vocais e a glote começa a se abrir. Após a abertura, a elasticidade dos tecidos das pregas vocais forçará as pregas vocais a inverter o movimento de abertura para fechamento^(2)^.

Desta forma, o treinamento e o uso adequado da musculatura respiratória influenciam na produção vocal eficiente, pois proporciona o fluxo aéreo necessário para que haja a pressão subglótica adequada para a vibração das pregas vocais. Para o treinamento da musculatura respiratória, existe uma diversidade nas abordagens de tratamento na clínica vocal, mas seus efeitos nas medidas aerodinâmicas ainda são escassos^(3)^.

Buscando-se melhorar a eficiência fonatória, fonoaudiólogos têm utilizado exercitadores e incentivadores respiratórios, para trabalhar as musculaturas inspiratória e expiratória, para além do trabalho específico da musculatura laríngea^(4-9)^. Isso porque tais recursos proporcionam aumento da capacidade pulmonar, gerando maior ativação do diafragma e músculos intercostais^(10)^.

Os exercitadores e incentivadores respiratórios são dispositivos que estimulam a respiração com base no esforço muscular. No caso dos exercícios inspiratórios com exercitadores e incentivadores respiratórios, eles incentivam uma inspiração profunda, com expansão máxima dos pulmões e, assim, auxiliam na atividade muscular, principalmente do diafragma e dos demais músculos intercostais responsáveis pelo movimento respiratório. Por tratar-se de músculos, eles podem ser treinados para a melhora da força e da resistência à fadiga^(11)^.

A literatura tem apontado que o treinamento inspiratório com exercitadores e incentivadores respiratórios é capaz de aumentar os tempos máximos de fonação, além de aumentar a capacidade de armazenamento de ar^(4)^. O aumento da força muscular respiratória a partir do treinamento com incentivadores também já é descrito, no entanto, seus efeitos nas medidas aerodinâmicas e nas características vocais dos sujeitos ainda não são claras^(5)^. Um recente estudo de revisão sistemática concluiu que ainda faltam evidências acerca da eficácia das intervenções respiratórias nos resultados vocais, destacando-se a importância de se compreender o mecanismo de ação dos exercícios e seus possíveis impactos na produção da voz^(10)^.

Durante a produção vocal, os músculos envolvidos na respiração trabalham em conjunto para fornecer o fluxo de ar necessário para produzir o som. Acredita-se que os estudos do treinamento respiratório, bem como os efeitos dele na voz são promissores para a Clínica Fonoaudiológica. A presente pesquisa pretende contribuir para o esclarecimento sobre os efeitos do treinamento muscular inspiratório com exercitador e incentivador respiratório na voz, para que seja possível se pensar na relevância e/ou viabilidade de sua introdução no processo de reabilitação dos distúrbios vocais e/ou no aprimoramento de vozes profissionais.

Partindo-se da hipótese de que os exercícios inspiratórios com exercitadores e incentivadores respiratórios poderiam impactar positivamente na voz, o objetivo do presente estudo foi avaliar o efeito imediato do exercício inspiratório com exercitador e incentivador respiratório nos dados perceptivo-auditivos, acústicos e aerodinâmicos de mulheres sem queixas vocais.

## MÉTODO

Trata-se de estudo de Intervenção do tipo “antes e após”.

A pesquisa foi aprovada pelo Comitê de Ética em Pesquisa com Seres Humanos com parecer de número 5.594.862.

### Casuística

A amostra foi constituída por conveniência, e a população alvo do estudo foi um grupo composto por mulheres sem queixas vocais. Os sujeitos foram controles deles mesmos.

A coleta de dados ocorreu no Laboratório de Voz da instituição de origem, em horários previamente estabelecidos com as mulheres participantes da pesquisa.

#### Critérios de inclusão

Foram incluídas mulheres sem queixas vocais, com idades acima de 18 anos, estudantes de cursos de graduação, que aceitaram participar voluntariamente das etapas da pesquisa e assinaram o Termo de Consentimento Livre e Esclarecido (TCLE).

#### Critérios de exclusão

Foram excluídas as mulheres com queixas vocais, que apresentaram pontuação igual ou superior a 5 no Índice de Triagem de Distúrbio de Voz (ITDV)^(12)^, tabagistas, que realizam ou realizaram tratamento vocal ou cirurgia de laringe, que autorrelataram perdas auditivas, doenças hormonais, gástricas, pulmonares, neurológicas e/ou psiquiátricas prévias, que apresentavam sinais e sintomas de alterações de vias aéreas no dia da coleta de dados da pesquisa, e as que não participaram de alguma etapa da pesquisa.

### Cálculo amostral

O cálculo do tamanho da amostra foi realizado por teste de hipóteses para o Teste de Duas Proporções a partir de um estudo semelhante que analisou o efeito do incentivador respiratório Shaker Plus antes e após três minutos de exercício. Os parâmetros fixos adotados para o teste foram α de 5%, β de 20% e K de 80%.

α: Erro α (Nível de significância) é a probabilidade de se estar errado ao aceitar a Hipótese Alternativa (H1) quando se utiliza um teste de hipótese na análise dos dados.

β: Erro β é a probabilidade de se estar errado ao se rejeitar a Hipótese Alternativa (H1) quando se utiliza um teste de hipótese na análise dos dados.

K (n pré/n pós): É a proporção do tamanho da amostra que se deseja entre o grupo pré e pós.

Para estimar a variabilidade da intervenção foram comparadas as proporções de melhora e de piora do grau geral de qualidade vocal entre os momentos pré e pós três minutos de intervenção para a amostra de contagem. Foram utilizados os valores de proporção de piora (p1=0,43) e de proporção de melhora (p2=0,07). O tamanho amostral calculado foi de 20 participantes no grupo pré e no grupo pós.

### Amostra

A coleta de dados ocorreu com 28 mulheres. Destas, 3 foram excluídas por apresentarem pontuação igual ou superior a 5 no Índice de Triagem de Distúrbio de Voz (ITDV)^(12)^.

Assim sendo, participaram do estudo 25 mulheres, com idades entre 18 e 34 anos e média de 21,04 anos de idade, o ITDV das mulheres incluídas no estudo teve pontuação média de 1 ponto, mínimo 0 e máximo de 3 pontos. Quanto à caracterização vocal, as participantes apresentavam grau geral de desvio vocal médio de 40 pontos na emissão de vogal e 32 pontos na emissão de números (EAV). Tinham, ainda, média de F_0_ de 214Hz e valores medianos de jitter% de 0,4, shimmer% 2,58, HNR 20,93, CPPs 14,81 e AVQI 1,68 e ABI 2,92.

### Procedimentos realizados

Foram realizados os seguintes procedimentos:

1. Assinatura do Termo de Consentimento Livre e Esclarecido;
2. Preenchimento de questionário acerca de dados de identificação e queixas vocais;
3. Aplicação do protocolo Índice de Triagem de Distúrbio de Voz (ITDV)^(12)^.
4. Gravação das seguintes amostras vocais^(1)^:
  a. Vogal sustentada /a/;
  b. Contagem dos números de 1 a 20;
  c. TMF: emissões sustentadas de /a/, /i/, /u/, /s/, /z/ e contagem de números em uma única expiração;

A coleta das amostras vocais foi realizada em laboratório de voz, utilizando-se microfone unidirecional da marca *Shure* modelo SM58, interface de áudio M Audio Fast Track, posicionado frontalmente à boca, numa distância aproximada de cinco centímetros. Todos os participantes foram orientados a manterem-se sentados, com o tronco ereto e costas apoiadas na cadeira, braços relaxados, mãos apoiadas nas pernas e pés apoiados no chão. A gravação foi feita pelo *software* Audacity, em taxa de amostragem de 44.100 Hz, canal mono em 16 Bit.

Por meio da gravação da voz foi realizado o Julgamento Perceptivo-Auditivo (JPA), a análise acústica vocal e a mensuração das medidas aerodinâmicas da emissão.

As participantes foram orientadas a realizar as emissões em frequência e intensidade habituais. Embora não tenha sido feito controle da pressão sonora por meio de medição em decibels (dB), a janela da entrada de áudio foi monitorada durante a gravação, de modo que o sinal preenchesse toda a faixa entre -0.5 e 0.5, sem ultrapassar a referida faixa, evitando-se saturação.

O treinamento respiratório por meio de incentivador inspiratório foi realizado da seguinte maneira:

d. Execução de uma série de 30 repetições em inspiração, com Exercitador e Incentivador respiratório Respiron® Classic^(13)^, obedecendo à seguinte orientação:

i. Inspire pela boca até elevar as três esferas do Respiron® Classic, mantendo-as elevadas pelo máximo de tempo possível, cuidando para não elevar excessivamente a musculatura acessória.

e. O exercício foi realizado na posição correspondente ao esforço zero, que tem nível de graduação zero, um, dois e três.

Imediatamente após a realização do exercício com o exercitador e incentivador respiratório, foi repetida a gravação da voz de acordo com a etapa 4.

### Julgamento perceptivo-auditivo

O julgamento perceptivo-auditivo (JPA) analisou o grau geral do desvio vocal da vogal sustentada /a/ e da contagem dos números de 1 a 11 pela Escala de Desvio Vocal^(14)^. Foi utilizada a escala analógico-visual (EAV) de 100 pontos, sendo que a extremidade à esquerda significa nenhum desvio e à direita, máximo desvio^(14)^.

A juíza, fonoaudióloga especialista em voz, com experiência clínica de 20 anos, recebeu uma pasta com as vozes aos pares, sem que soubesse qual era o momento pré e qual era o momento após. Foi realizada a análise de concordância interna da juíza, pelo teste estatístico Coeficiente de Correlação Intraclasse (CCI), repetindo-se 20% da amostra, de modo aleatório. Após análise pelo CCI, verificou-se que os pares tiveram concordância entre si, todos com respostas acima de 0,9, o que determina excelente concordância.

### Análise acústica da voz

Para a realização da análise acústica vocal foi utilizado o programa *PRAAT*, sendo extraídas as seguintes medidas pela emissão da vogal sustentada /a/:

- Frequência fundamental média (F_0_): corresponde ao número de ciclos glóticos produzidos pelas pregas vocais em um segundo^(1)^;
- *Jitter:* corresponde à variabilidade da F_0_ em curto prazo, considerando os ciclos glóticos, que estão um na sequência do outro. Mensura o quanto um ciclo é diferente do seu antecessor de acordo com a perturbação de frequência^(1)^;
- *Shimmer:* corresponde à perturbação de amplitude em curto prazo, que indica pequenas variações no controle da saída do ar pelas PPVV e na intensidade da emissão^(1)^;
- *Harmonic-to-Noise Ratio* (HNR): fornece informações complementares entre a relação do componente harmônico e a quantidade de ruído sonoro na amostra vocal^(1)^;
- *CPPS*: mede o grau de periodicidade do sinal vocal acima dos ruídos presentes nas emissões de vogal sustentada e de fala encadeada, fornecendo seus resultados em decibel. A medida produz uma melhora eminente na precisão da análise das vozes desviadas^(15)^;

Também foram extraídas por meio da emissão da vogal /a/ associada à contagem dos números de 1 a 11 as seguintes medidas:

- *Acoustic Voice Quality Index (AVQI):* quantifica a intensidade de desvio da qualidade vogal^(16-17)^;
- *Acoustic Breathiness Index (ABI)*: fornece informações para triagem e acompanhamento do paciente quanto à qualidade vocal soprosa^(18)^.

### Medidas aerodinâmicas da emissão

Para a realização das medidas aerodinâmicas, foi utilizado o programa Audacity. A análise das medidas foi realizada por uma pesquisadora que não participou de nenhuma das etapas anteriores da pesquisa. Foi coletado o Tempo Máximo de Fonação (TMF), parâmetro com que se obtêm medidas respiratórias e permite uma investigação quantitativa e qualitativa da fonação^(1,19)^, foram utilizadas as medidas de:

- /a/, /i/, /u/, /s/, /z/, e contagem de números em única expiração^(1,19)^;
- Relação s/z – extraída a partir da emissão sustentada das fricativas mediais surdas e sonoras /s/ e /z/. A partir disso foi realizada a proporção entre elas, dividindo-se o tempo do /s/ pelo do /z/. Com essa medida pode-se obter a relação entre as forças aerodinâmicas pulmonares e mioelásticas da laringe^(1,19)^.

Os resultados foram comparados analisando-se as amostras dos registros do Grupo de estudo antes da realização do exercício inspiratório (GE1) e do Grupo de estudo após a realização do exercício (GE2). Estes foram tabulados e utilizados para a análise estatística, na busca por respostas das seguintes hipóteses:

H0: não há diferenças entre o julgamento perceptivo-auditivo, a análise vocal acústica, e as medidas aerodinâmicas da emissão antes e após a realização imediata do exercício com incentivador inspiratório.

H1: não há igualdade entre o julgamento perceptivo-auditivo na análise vocal acústica e as medidas aerodinâmicas da emissão antes e após a realização imediata do exercício com incentivador inspiratório.

Hipotetiza-se que o trabalho com a musculatura respiratória pode melhorar aspectos da mobilidade muco-ondulatória das pregas vocais, por meio da modificação do fluxo transglótico e da pressão subglótica após o treinamento inspiratório, assim sendo o grau geral do desvio vocal, medidas acústicas e aerodinâmicas foram selecionadas para a avaliação multidimensional da voz após a realização do exercício inspiratório com exercitador e incentivador respiratório.

### Variáveis do estudo

As variáveis dependentes (desfechos) do estudo são o julgamento perceptivo-auditivo, as medidas acústicas e aerodinâmicas da emissão. A variável independente foi a realização do exercício com exercitador e incentivador respiratório.

### Análise de dados

Os resultados foram tabulados em planilha do Microsoft Excel e submetidos à análise estatística.

As medidas de tendência central e de dispersão foram expressas em médias e desvio padrão (média + DP) para as variáveis contínuas de distribuição simétrica e em medianas, valores mínimo e máximo (mediana, mínimo – máximo) para as de distribuição assimétrica.

Os dados foram analisados utilizando-se o *software Statistica for Windows*. A normalidade das variáveis foi testada por meio do teste Shapiro Wilk (p<0,05), sendo que todas tiveram distribuição normal, exceto a medida TMF /a/, que teve distribuição não normal. Para comparar os resultados utilizou-se o teste paramétrico t de *Student* para variáveis dependentes para as medidas com distribuição normal e o teste de Wilcoxon para única medida não normal. Para todos os testes foi considerado um nível mínimo de significância de 5% (p > 0,05).

## RESULTADOS

Participaram do estudo 25 mulheres com média de idade de 21,04 anos, mínimo de 18 anos e máximo de 34 anos. Todas eram estudantes universitárias.

Na Tabela 1 estão apresentados os resultados referentes à distribuição dos valores do julgamento perceptivo-auditivo do grau geral do desvio vocal antes e após o exercício inspiratório com exercitador e incentivador respiratório, para a emissão da vogal sustentada /a/ e da contagem dos números. Não houve diferenças entre as emissões antes e após a realização do exercício. É importante mencionar que os valores médios apresentados pelas participantes, tanto no pré quanto no pós, estavam dentro da variabilidade normal para a amostra de números e como desvios discretos para a amostra de vogal^(14)^.

**Tabela 1:** Distribuição dos valores do julgamento perceptivo-auditivo do grau geral do desvio vocal do vogal /a/ e da contagem de números antes e após realização do exercício com incentivador inspiratório (n=25)

		Média	Mediana	Mín.	Máx.	Desvio Padrão	p
**Vogal /a/**	Pré	40,00	39	23	53	9,34	0,654
	Pós	40,76	40	25	56	9,43	
**Contagem de Números**	Pré	32,00	32	10	54	12,32	0,221
	Pós	29,28	29	13	51	10,73	

Test t de student para variáveis dependentes

Na Tabela 2 estão apresentados os resultados referentes à distribuição dos valores acústicos antes e após o exercício inspiratório com exercitador e incentivador respiratório. Não houve diferenças entre as análises antes e após a realização do exercício.

**Tabela 2:** Distribuição dos valores acústicos antes e após realização do exercício com incentivador inspiratório (n=25)

		Média	Mediana	Mín.	Máx.	Desvio Padrão	p
**F0 (%)**	Pré	214,56	225,99	111,95	250,17	30,72	0,513
	Pós	219,26	217,21	157,16	276,25	24,91	
***Jitter (%)***	Pré	0,40	0,40	0,18	0,60	0,12	0,890
	Pós	0,40	0,34	0,17	0,99	0,21	
***Shimmer (%)***	Pré	2,58	2,44	1,12	5,72	0,92	0,566
	Pós	2,46	2,13	1,04	5,25	1,09	
***Shimmer (dB)***	Pré	0,22	0,21	0,09	0,49	0,08	0,616
	Pós	0,21	0,19	0,09	0,46	0,09	
**HNR (%)**	Pré	20,93	21,22	16,06	28,56	2,79	0,925
	Pós	20,98	21,43	15,80	26,16	2,77	
**CPPS (%)**	Pré	14,81	14,58	12,29	17,87	1,49	0,322
	Pós	15,09	15,31	12,15	17,65	1,59	
**AVQI (%)**	Pré	1,68	1,57	0,14	3,03	0,78	0,532
	Pós	1,75	2,05	0,05	3,66	0,87	
**ABI (%)**	Pré	2,92	2,83	1,7	5,08	0,8	0,889
	Pós	2,94	2,91	1,8	5,25	0,81	

Test t de student para variáveis dependentes

Na Tabela 3 estão apresentados os resultados referentes à distribuição das medidas aerodinâmicas antes e após a realização do exercício inspiratório com exercitador e incentivador respiratório. Houve diferença entre os valores de TMF /s/ antes e após a realização do exercício inspiratório.

**Tabela 3:** Distribuição dos valores dos tempos máximos de fonação antes e após realização do exercício com incentivador inspiratório (n=25)

		Média	Mediana	Mín.	Máx.	Desvio Padrão	p
**TMF /a/**	Pré	14,12	13	10	28	4,35	0,930^1^
	Pós	13,8	13	10	21	2,95	
**TMF /i/**	Pré	13,72	14	8	23	3,27	0,262
	Pós	14,16	13	10	24	3,36	
**TMF /u/**	Pré	13,68	14	7	22	3,27	0,320
	Pós	14,28	14	9	23	3,27	
**TMF /s/**	Pré	12,84	12	5	24	4,37	0,008^*^
	Pós	14,56	15	6	22	4,26	
**TMF /z/**	Pré	13,72	13	6	28	4,70	0,348
	Pós	14,40	13	8	25	4,67	
**TMF contagem de números**	Pré	17,04	16	10	29	4,72	0,232
	Pós	17,76	17	11	27	4,24	
**Relação s/z**	Pré	0,971	0,93	0,52	1,72	0,307	0,183
	Pós	1,037	1,0	0,64	1,70	0,266	

1 Test t de Student para variáveis dependentes;

Teste de Wilcoxon

* p<0,05

## DISCUSSÃO

A avaliação do efeito imediato de uma técnica vocal busca compreender as modificações fisiológicas do mecanismo fonatório^(20)^. A presente pesquisa, buscou investigar os efeitos imediatos do exercício inspiratório com exercitador e incentivador respiratório em mulheres sem queixas vocais. A escolha por mulheres sem queixas vocais buscou compreender o efeito do exercício primeiramente em mulheres vocalmente saudáveis ou com leve desvio vocal para subsidiar estudos futuros em sujeitos disfônicos. Este estudo também busca evidências que possam auxiliar o clínico na seleção de técnicas mais eficientes para resultados imediatos. Entende-se que os músculos envolvidos na respiração trabalham em conjunto para fornecer o fluxo de ar necessário para produzir a voz, e que o treinamento desta musculatura pode modificar o fluxo de ar transglótico e a pressão de ar, gerando impacto vocal.

A amostra selecionada para o estudo foi constituída por mulheres sem queixas vocais, que passaram na triagem vocal pelo ITDV^(12)^, e tiveram classificação vocal por meio de julgamento perceptivo-auditivo do grau geral do desvio vocal pela Escala de Desvio Vocal (EDV)^(14)^. A classificação do desvio vocal foi compatível com a de vozes saudáveis a levemente alteradas para a contagem de números (entre 0 e 35,5 mm)^(14)^ e de leve a moderada para a emissão da vogal sustentada, (entre 35,5 mm e 50,5 mm)^(14)^, sem diferenças antes e após a realização do exercício com o incentivador inspiratório. As diferentes classificações entre as amostras vocais coletadas, vogal sustentada e contagem de números, podem ter ocorrido devido aos ajustes musculares necessários entre fonte e filtro nas diferentes nas emissões. Acredita-se que o fato de ter havido desvios discretos na emissão de vogais não comprometa os resultados do estudo, já que os valores médios estavam muito próximos do limite da variabilidade normal. Além disso, desvios discretos não são incomuns em pessoas sem queixas vocais.

Observou-se que a realização do exercício inspiratório com exercitador e incentivador respiratório não foi capaz de gerar modificações na qualidade vocal imediatamente após a sua realização. De fato, tal exercício não tem ação laríngea direta. Assim sendo, a realização de exercícios inspiratórios com exercitador e incentivador respiratório não deve ser realizada com finalidade de se obter modificação da qualidade vocal de forma imediata, como por exemplo nos programas de aquecimento vocal. Técnicas vocais que obtiveram respostas positivas na qualidade vocal em estudos de efeitos imediatos são mais indicadas para este fim. No entanto, acredita-se que dados perceptivo-auditivos devam ser mais bem explorados, sobretudo em pesquisas com treinamentos respiratórios mais longos (semanas, meses), partindo-se da hipótese de que aumentos na pressão subglótica poderiam passar a ter efeitos na coaptação e vibração das pregas vocais e, consequentemente, na estabilidade da voz.

Como parte da avaliação multidimensional da voz foi realizada a avaliação acústica que permite a mensuração das propriedades do sinal vocal de forma quantitativa, permitindo uma análise objetiva da voz^(21)^. A análise acústica é muito utilizada na clínica vocal fonoaudiológica, por meio das medidas de F0, *jitter, shimmer*, HNR (*Harmonic noise ratio*), CPPS, AVQI, entre outras^(16-17,22-23)^.

No que diz respeito à avaliação acústica vocal, assim como no julgamento perceptivo-auditivo, não foram observadas diferenças na comparação dos momentos antes e após a realização do exercício inspiratório com o exercitador e incentivador respiratório. Este exercício trabalha principalmente a musculatura respiratória, como o músculo diafragma e músculos intercostais.

Há a possibilidade de que com o treinamento por mais tempo haja modificações destes parâmetros. A medida que mais variou foi a F_0_, com diminuição, porém não significativa. Tal variação pode estar relacionada ao abaixamento da laringe que ocorre durante a realização do exercício. Mais estudos são necessários para investigar se este e outros parâmetros podem sofrer modificações com a realização de exercícios inspiratórios por períodos mais longos. Não foram encontrados estudos que investigassem as medidas acústicas vocais relacionadas à exercícios com incentivador inspiratório.

Quanto às medidas aerodinâmicas avaliadas por meio dos tempos máximos de fonação, foi possível observar que houve modificações significativas nos valores de TMF do /s/ após a realização do exercício com o incentivador inspiratório. O TMF se trata de uma medida acústica capaz de analisar as forças aerodinâmicas pulmonares e mioelásticas da laringe^(1,24-25)^.

O fonema /s/ é não vozeado porque não utiliza da vibração das pregas vocais. Pode-se evidenciar com esta medida o controle aerodinâmico da emissão com a saída do ar expiratório de maneira quantitativa, constatando assim o desempenho do nível respiratório na fonação^(1,19,24)^. Tal resultado evidencia que a prática do exercício com incentivador respiratório pode proporcionar aumento da capacidade pulmonar.

As evidências de tratamento vocal sobre o tempo máximo fonatório (TMF) foram quantificadas por meio da abordagem estatística de uma meta-análise. Foram considerados estudos que eram relatos de ensaios clínicos randomizados controlados (ECR) avaliando a eficácia de um tratamento específico de fonoterapia utilizando TMF como medida de desfecho em participantes adultos com distúrbios vocais. Os autores identificaram que a única intervenção efetiva com efeito significativo foi o exercício de função vocal (FV) e concluíram que ele melhorou efetivamente o TMF do pré para o pós-tratamento em comparação com outras intervenções vocais comparadas. Os autores sugerem ainda que mais estudos de intervenção de alta qualidade com grandes tamanhos amostrais, medidas multidimensionais e grupos homogêneos de disfonia são necessários para apoiar a prática baseada em evidências em laringologia^(3)^.

A influência da posição corporal e do número de emissões nos resultados dos tempos máximos de fonação (TMF) de adultos sem queixa vocal foram analisados em estudo anterior com sessenta sujeitos, sendo eles 30 homens e 30 mulheres sem queixas vocais. Os sujeitos foram orientados a permanecer na posição ortostática ou sentada, de acordo com a ordem de coleta selecionada. Os 30 primeiros sujeitos foram avaliados pela ordem número 1 (sentado e ortostático) e os outros 30 sujeitos pela ordem número 2 (ortostático e sentado), com intervalo de 5 minutos entre as emissões nas duas posições. Foram coletados os TMF das vogais /a/, /i/, /u/, fricativas /s/ e /z/ e números. Em seguida, os sujeitos foram orientados a realizar as mesmas emissões citadas acima, porém em outra posição (ortostática ou sentada). Não houve diferença entre os TMF obtidos nas duas posições. Todos os TMF do sexo masculino foram maiores que os do sexo feminino. Em relação ao número de emissão, houve diferenças para ambos os sexos no TMF da vogal /a/ e na contagem numérica. Os autores concluíram que de modo geral que não há influência da posição corporal nos resultados das medidas temporais de voz, mas o sexo e o número de emissões influenciam no resultado dos TMF^(19)^.

Um estudo com cinco adultos saudáveis identificou que após o treinamento durante quatro semanas com o incentivador respiratório Respiron®, modelo Classic Nível Médio, houve melhora nas médias finais dos TMF. Os autores sugeriram que isso aconteceu em razão de que o uso dos incentivadores respiratórios proporcionou aumento na capacidade de armazenamento de ar, portanto o fluxo aerodinâmico maior proporcionou controle vocal e capacidade de emissão maiores^(4)^.

Outro estudo buscou determinar e avaliar os efeitos do Exercício de Respiração Diafragmática na função respiratória e sustentação vocal entre vocalistas aparentemente saudáveis. Os autores observaram que o exercício respiratório teve efeito direto sobre a função pulmonar e efeito indireto sobre o tempo máximo de fonação dos vocalistas, visto que houve melhora significativa nas funções respiratórias e os valores respiratórios correlacionaram-se significativamente com os valores do TMF. Verificaram também que alterações nas funções respiratórias acarretaram alterações no tempo máximo de fonação, pois cantores masculinos da pesquisa apresentaram mais alterações na função respiratória do que as cantoras, que apresentaram melhores resultados no TMF^(25)^.

A literatura ainda apresenta escassez de estudos relacionados aos efeitos do exercício inspiratório com exercitador e incentivador respiratório na voz. Foram encontrados outros estudos utilizando o equipamento Respiron® Classic, no entanto de forma invertida, para trabalhar a musculatura expiratória. O exercitador e incentivador respiratório “Respiron® Classic- NCS” foi desenvolvido visando o fortalecimento da musculatura inspiratória, entre eles o principal músculo, o diafragma. Dessa forma, de acordo com as instruções de uso do equipamento, não se recomenda o uso do dispositivo de forma invertida e por este motivo tais estudos não foram citados.

Uma limitação encontrada neste trabalho foi relativa à adesão dos homens ao estudo. Assim como também foi evidenciado em outro estudo com exercícios respiratórios, porém de expiração, no qual as autoras apontaram que houve dificuldades na participação de homens, o que fez com que o estudo fosse realizado também apenas com mulheres^(8)^. Aponta-se, ainda, como limitação, a ausência da autoavaliação das participantes acerca de possíveis efeitos do exercício na voz. Esclarece-se que tal procedimento não foi incluído pelo fato de não haver instrumentos de autoavaliação disponíveis especificamente para este fim. Diante disso, entendemos que a aplicação de protocolos de autoavaliação existentes, porém com outras finalidades, não trariam dados relevantes para o presente estudo. Sinalizamos a importância do desenvolvimento de instrumentos sensíveis para identificar a percepção vocal do sujeito/paciente acerca dos efeitos imediatos de técnicas vocais.

Sugere-se que novos estudos com exercícios inspiratórios sejam realizados para se testar os efeitos imediatos em diferentes populações como homens sem queixas vocais, e mulheres e homens disfônicos, bem como com outros meios de investigação como a Espirometria para fins fonoaudiológicos e com maior tempo de utilização para verificar os efeitos do treinamento muscular durante semanas consecutivas.

## CONCLUSÃO

O aumento do tempo máximo de fonação do /s/ em mulheres sem queixas vocais demonstra a eficácia imediata da realização do exercício inspiratório com exercitador e incentivador respiratório na capacidade pulmonar, demonstrando aprimoramento do controle aerodinâmico com melhor desempenho respiratório à fonação.

Nas demais avaliações vocais multidimensionais realizadas no presente estudo, como no julgamento perceptivo-auditivo e nas medidas acústicas, o exercício inspiratório não causou modificações imediatas.
